# Paris Hospitals

**Published:** 1919-01

**Authors:** 


### PARIS HOSPITALS

Medical officers, who have had the opportunity to visit Paris, have made frequent requests from the American Red Cross regarding various clinics and other hospital activities that are open to them in the great French center of medicine and surgery. In order to supply this information Captain T. D. Boulanger, Liaison Officer in the Department of Medical Research of the American Red Cross, has visited most of the leading hospitals in Paris, and has collected data concerning them and the work that is being done by members of their staffs. He has prepared for publication a list of the hospitals, giving their locations, the character of the work done in them, and the names of their staffs. It should be understood that the staffs of the hospitals during the war, and at this time, are necessarily not the same as in pre-war days, and as they may be after the demobilization of the many distinguished Parisian physicians and surgeons now in the French Army. It is, therefore, likely that many changes will be made in the staffs of the Paris hospitals within the next few months.

The members of the French medical profession have been extremely courteous and cordial to visiting American physicians and surgeons. Indeed, they express regret that more of our medical men have not visited their hospitals and clinics. Some of the finest and best conducted hospitals in the world are in Paris, and the medical officer who comes to this city and does not visit the clinics is missing an opportunity which he will regret on his return to work in civil life.

Captain Boulanger's article on the Paris hospitals is published in this number of *War Medicine*. Reprints of it will be made, which will serve as an interesting guide to medical officers wishing to avail themselves of the opportunity to see something of French clinics and of hospital administration in Paris. Copies of this brochure on Paris hospitals may be obtained by application to the Department of Medical Research, American Red Cross, 2 Place de Rivoli, Paris.

